# Author Correction: Activation of subnanometric Pt on Cu-modified CeO_2_ via redox-coupled atomic layer deposition for CO oxidation

**DOI:** 10.1038/s41467-020-19663-3

**Published:** 2020-11-12

**Authors:** Xiao Liu, Shuangfeng Jia, Ming Yang, Yuanting Tang, Yanwei Wen, Shengqi Chu, Jianbo Wang, Bin Shan, Rong Chen

**Affiliations:** 1 State Key Laboratory of Digital Manufacturing Equipment and Technology, School of Mechanical Science and Engineering, Huazhong University of Science and Technology, 430074 Wuhan, Hubei People’s Republic of China; 2 State Key Laboratory of Materials Processing and Die and Mould Technology, School of Materials Science and Engineering, Huazhong University of Science and Technology, 430074 Wuhan, Hubei People’s Republic of China; 3 School of Physics and Technology, Center for Electron Microscopy, MOE Key Laboratory of Artificial Micro- and Nano-structures, and Institute for Advanced Studies, Wuhan University, 430072 Wuhan, Hubei People’s Republic of China; 4 General Motors Global Research and Development, Chemical Sciences and Materials Systems Lab, 3500 Mound Road, Warren, MI 48090 USA; 5 Institute of High Energy Physics, Chinese Academy of Sciences, 100049 Beijing, People’s Republic of China; 6 Present Address: Department of Chemical and Biomolecular Engineering, Clemson University, Clemson, SC 29634 USA

**Keywords:** Devices for energy harvesting, Electrical and electronic engineering, Applied physics

Correction to: *Nature Communications* 10.1038/s41467-020-18076-6, published online 25 August 2020.

This Article contains an error in Fig. 2a, where the figure legends of catalytic curves between support Pt_*n*_ catalysts (Pt_*n*_/CeCu and Pt_*n*_/Ce) and supported Pt_1_ catalysts (Pt_1_/CeCu and Pt_1_/Ce) were inadvertently reversed.

The error has been corrected in the PDF and HTML versions of the Article.

