# Supplementary material for: Activation of subnanometric Pt on Cu-modified CeO2 via redox-coupled atomic layer deposition for CO oxidation
Source: Nat Commun. 2020 Aug 25;11:4240. doi: 10.1038/s41467-020-18076-6 (PMC7447628; doi:10.1038/s41467-020-18076-6)
Supplement: Supplementary file 1 — Supplementary Information [file 41467_2020_18076_MOESM1_ESM.pdf]

Supplementary Information

Activation of subnanometric Pt on Cu-modified CeO_2 via redox-coupled atomic layer deposition for CO oxidation

Xiao Liu, Shuangfeng Jia, Ming Yang, Yuanting Tang, Yanwei Wen, Shengqi Chu, Jianbo Wang, Bin Shan,* and Rong Chen*

*Corresponding authors: rongchen@mail.hust.edu.cn (Rong Chen); bshan@mail.hust.edu.cn (Bin Shan).

Supplementary Figures

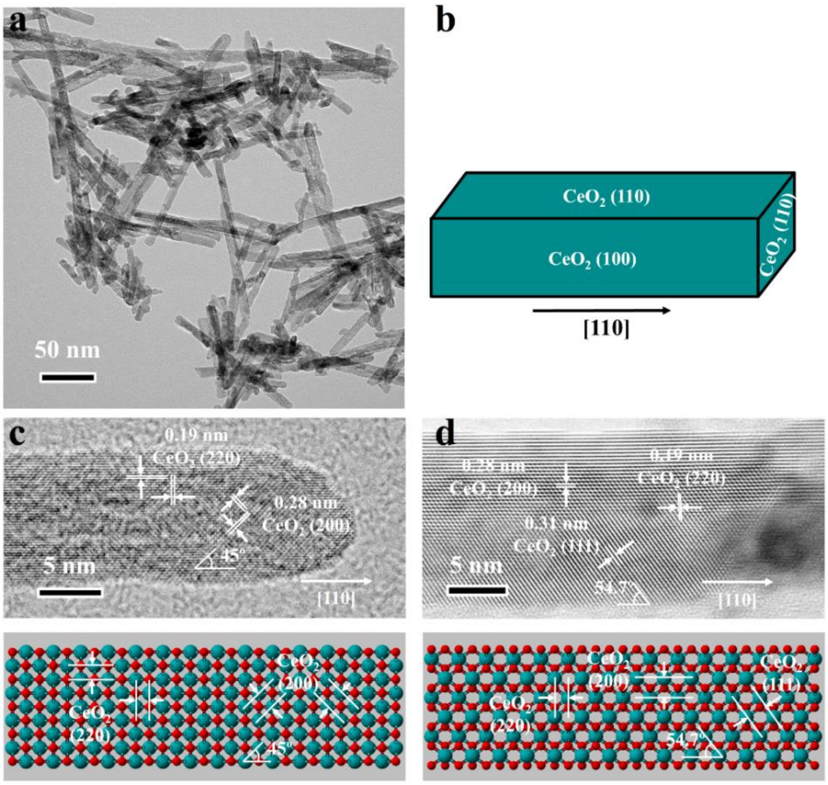

Supplementary Figure 1. Characterizations of CeO_2 nanorod's exposed facets. **a** transmission electron microscopy (TEM) image and **b** schematic diagram of as prepared CeO_2 nanorods. The high resolution TEM images in **c** and **d** show the CeO_2 nanorods are grown along [110] direction. The confirmed crystal planes of (220) and (200) based on the measured d-spacing, as well as their included angle of 45° imply the main exposed facets are (220) and (200), which agrees well with previous studies[1,2]. The observed CeO_2 (111) crystal plane exhibits an angle of 54.7° from the exposed (200) facet, agreeing well with the atomic model of CeO_2 nanorod.

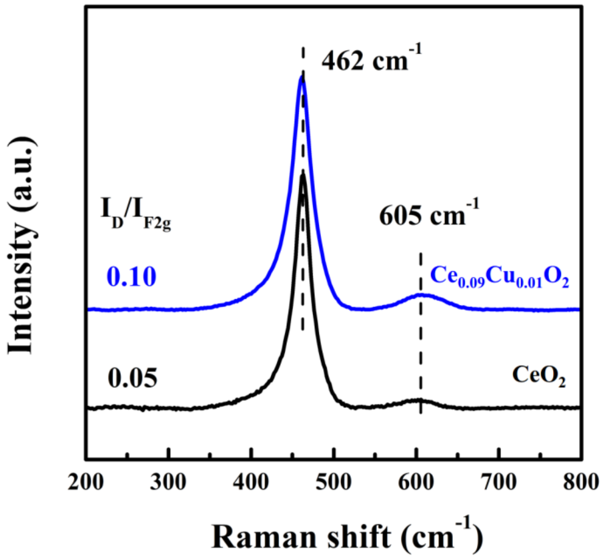

Supplementary Figure 2. Raman spectra of CeO_2 and $Ce_{0.99}Cu_{0.01}O_2$ supports. The ratios of I_D (defect-induced mode) and I_{F2g} (F_{2g} mode) have been labelled, which can imply the concentration of surface defect site.

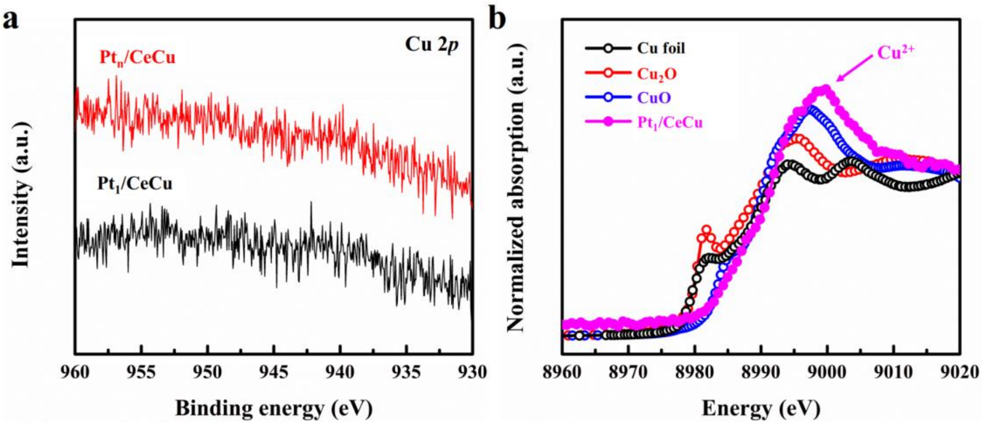

Supplementary Figure 3. Chemical state of Cu dopants in CeO_2 nanorods. **a** Cu 2*p* XPS spectra of Pt_1/CeCu and Pt_n/CeCu. **b** Normalized Cu K-edge X-ray absorption near edge structure (XANES) spectrum of Pt_1/CeCu in comparison to Cu foil, Cu_2O and CuO references.

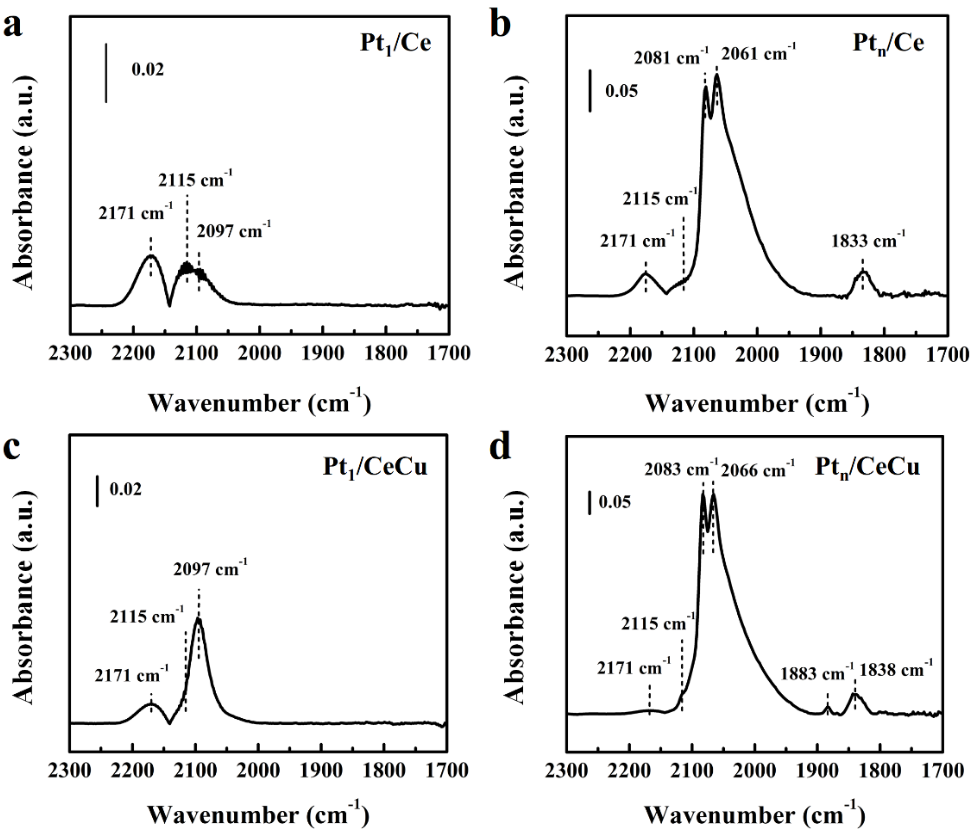

Supplementary Figure 4. Morphology characterizations of supported Pt catalysts. Diffuse reflectance infrared Fourier transform spectroscopy (DRIFTS) spectra of CO adsorption on **a** Pt_1/Ce, **b** Pt_n/Ce, **c** Pt_1/CeCu and **d** Pt_n/CeCu. The peaks at 2171 cm^{-1} and 2115 cm^{-1} are assigned to the stretching bands of gas phased CO molecules. The peak at 2097 cm^{-1} is assigned to CO adsorption on atomically dispersed Pt. The signals at the range of 2083 cm^{-1} ~ 1950 cm^{-1} are from linear bonded CO at the surface of Pt clusters and the signals near the 1833 cm^{-1} and 1883 cm^{-1} are from bridged bonded CO at the surface of Pt clusters[3,4].

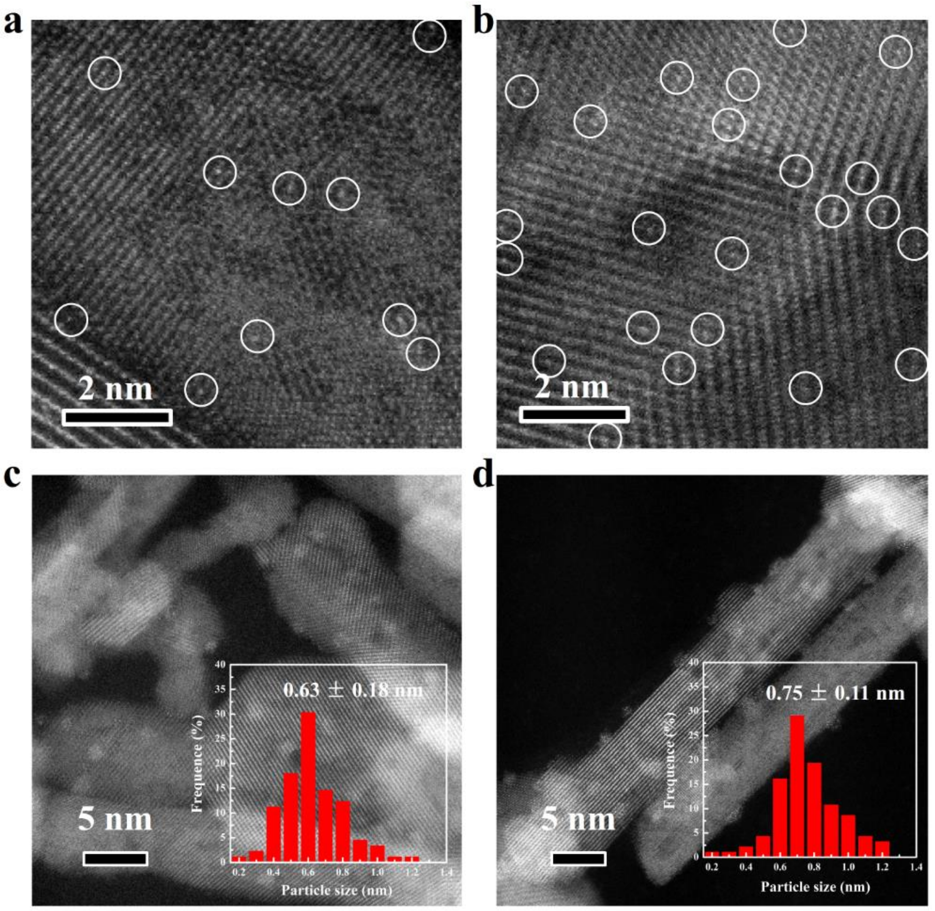

Supplementary Figure 5. Morphology characterizations of supported Pt catalysts. Representative aberration-corrected high-angle annular dark-field scanning transmission electron microscopy (HAADF-STEM) images of **a** Pt_1/Ce, **b** Pt_1/CeCu, **c** Pt_n/Ce and **d** Pt_n/CeCu catalysts. The numbers of Pt single atoms per unit area for Pt_1/Ce and Pt_1/CeCu are estimated to 0.24 Pt/nm^2 and 0.59 Pt/nm^2 based on the specific surface area of 79.4 m^2/g as reported in our previous study[5] and the mass loading of Pt clusters, which are consistent with that calculated based on the Pt atoms in the area with size of 8 × 8 nm^2. The size distributions are inserted and the average sizes of Pt clusters are 0.63 ± 0.18 nm and 0.75 ± 0.11 nm, respectively.

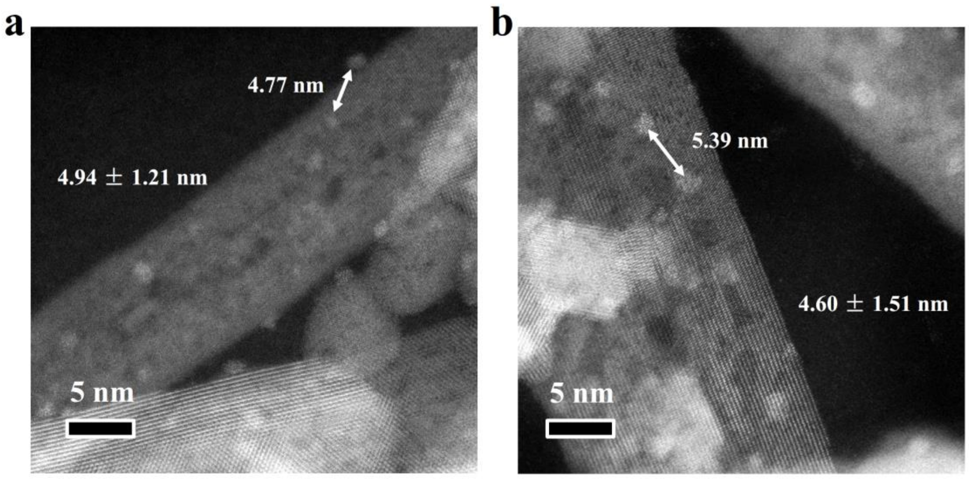

Supplementary Figure 6. Distribution of Pt clusters on CeO_2 nanorods. Representive HAADF-STEM images of **a** Pt_n/Ce and **b** Pt_n/CeCu. The average distances of Pt clusters in a CeO_2 nanorod are labelled.

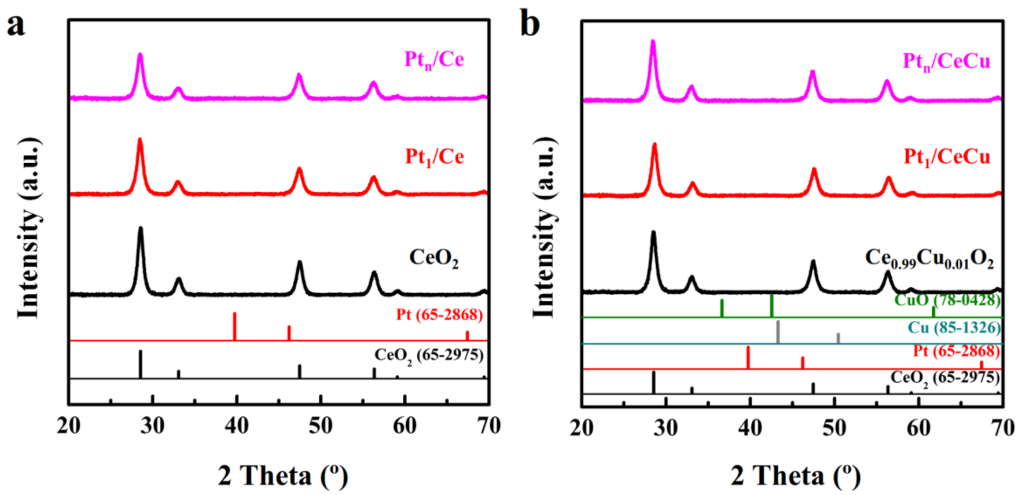

Supplementary Figure 7. Crystalline structures of prepared catalysts. XRD patterns of **a** CeO_2, Pt_1/Ce, Pt_n/Ce and **b** $Ce_{0.99}Cu_{0.01}O_2$, Pt_1/CeCu, Pt_n/CeCu. CeO_2 nanorod supports exhibit standard fluorite structure (JCPDS No. 65-2975). Cu dopants cannot change the crystalline structure of CeO_2 without CuO (JCPDS No. 78-0428) and Cu phase (JCPDS No. 85-1326). The diffraction peaks (JCPDS No. 65-2868) of highly dispersed Pt atoms and clusters cannot be detected for Pt_1/Ce, Pt_n/Ce, Pt_1/CeCu and Pt_n/CeCu catalysts.

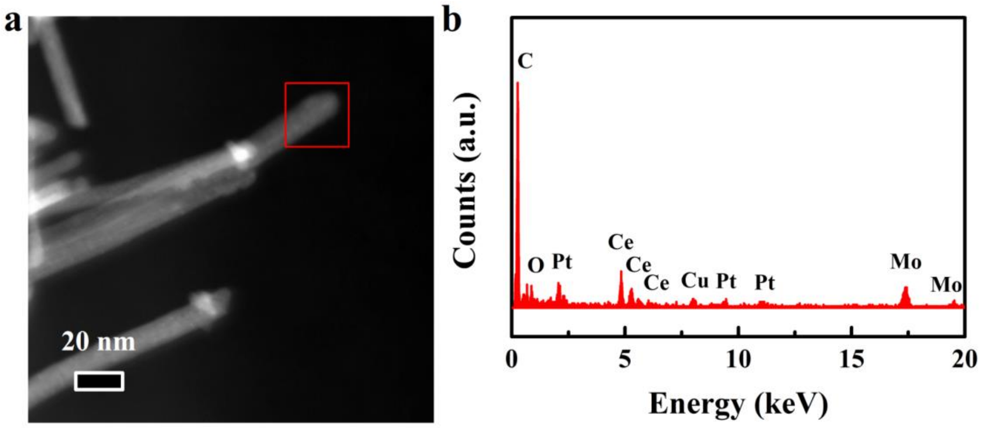

Supplementary Figure 8. Element analysis of Pt_n/CeCu catalyst. **a** STEM image and **b** energy dispersive spectroscopy (EDS) spectrum of Pt_n/CeCu catalyst. The presence of Mo element is due to Mo mesh served as supports instead of Cu mesh, which can avoid the interference during detecting Cu element in the catalyst.

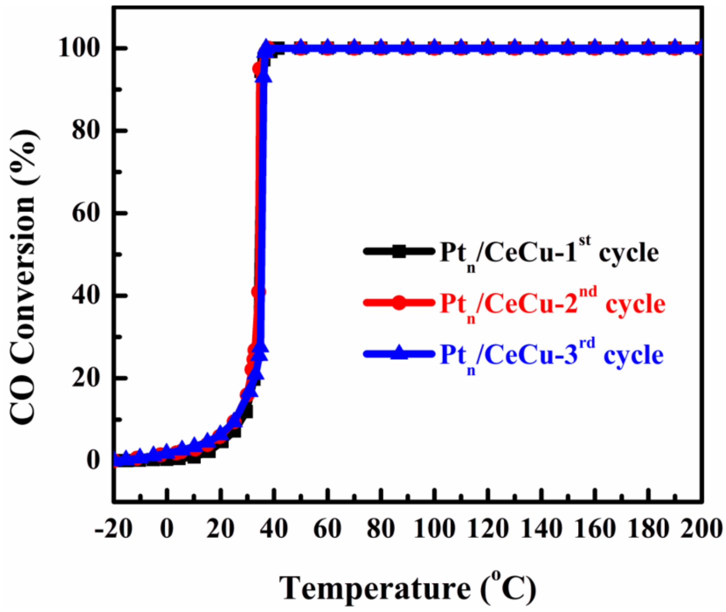

Supplementary Figure 9. Reproducibility test of prepared catalyst. CO conversion curves of Pt_n/CeCu catalyst for three cycles. The slightly shifts of CO conversion curves indicate the reproducibility of our catalytic results.

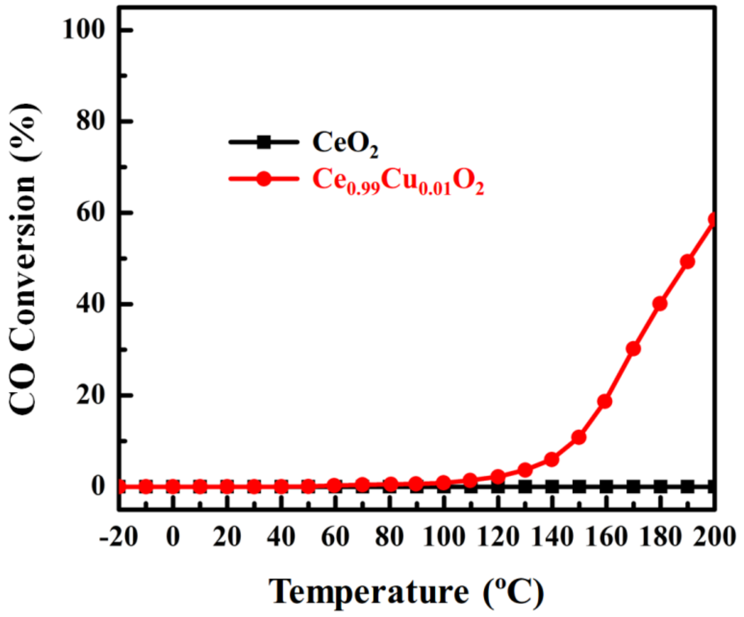

Supplementary Figure 10. Catalytic activities of oxide supports. CO conversion of CeO_2 and $Ce_{0.99}Cu_{0.01}O_2$ supports as a functional of reaction temperature.

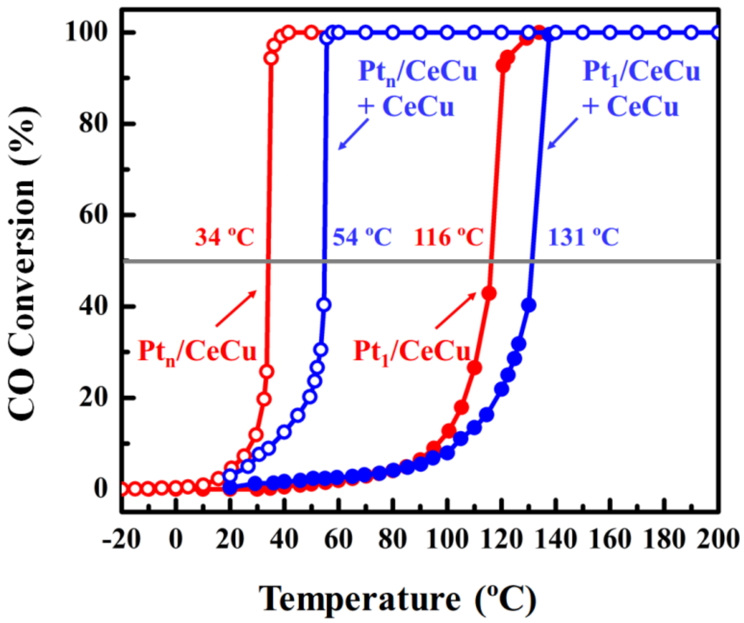

Supplementary Figure 11. Catalytic activities of prepared catalysts. CO conversion of Pt_n/CeCu and Pt_1/CeCu catalysts diluted with $Ce_{0.99}Cu_{0.01}O_2$ supports as a functional of reaction temperature. 20 mg Pt_n/CeCu or Pt_1/CeCu has been diluted with 30 mg $Ce_{0.99}Cu_{0.01}O_2$ supports to make sure the mass loading of Pt similar to Pt_n/Ce or Pt_1/Ce catalysts.

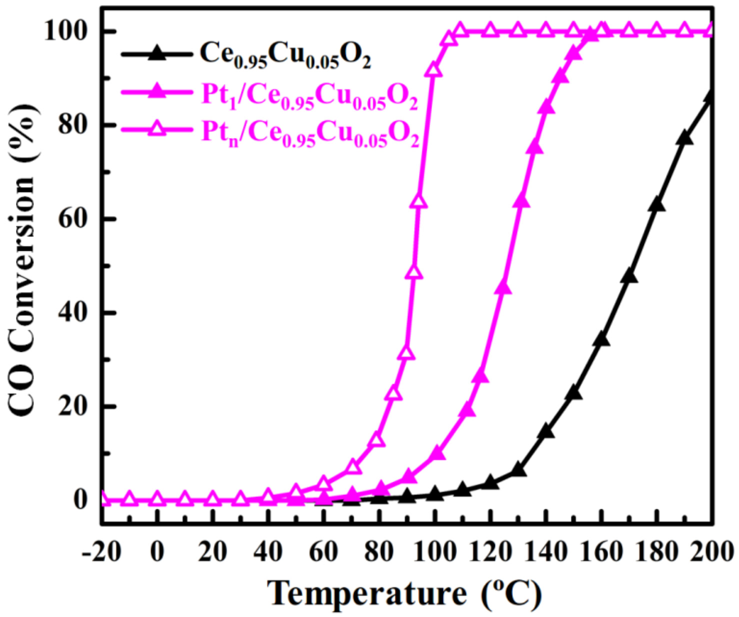

Supplementary Figure 12. Catalytic activities of prepared catalysts. CO conversion of $Ce_{0.95}Cu_{0.05}O_2$, $Pt_1/Ce_{0.95}Cu_{0.05}O_2$ and $Pt_1/Ce_{0.95}Cu_{0.05}O_2$ as a functional of reaction temperature. The Pt atomic layer deposition (ALD) processes of $Pt_1/Ce_{0.95}Cu_{0.05}O_2$ and $Pt_1/Ce_{0.95}Cu_{0.05}O_2$ are the same as that of Pt_1/CeCu and Pt_n/CeCu.

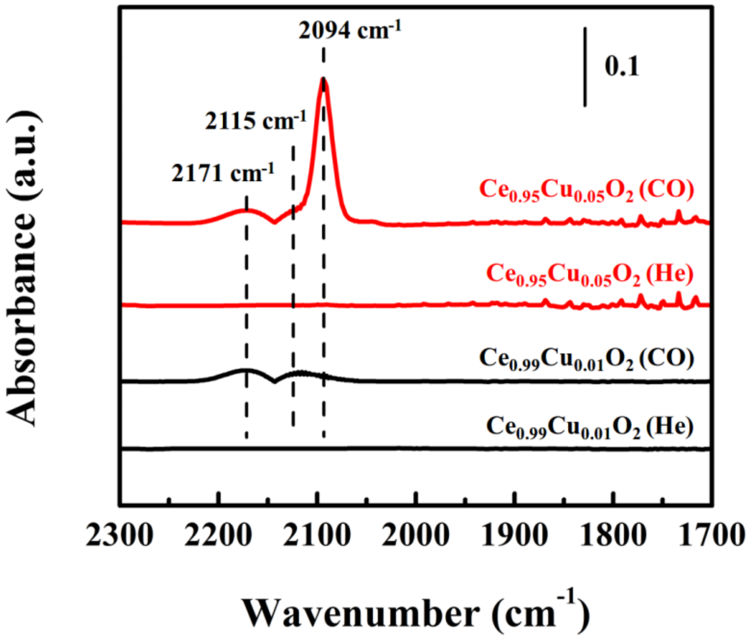

Supplementary Figure 13. Surface structures of Cu doped CeO_2 nanorods. *In situ* DRIFTS spectra of $Ce_{0.95}Cu_{0.05}O_2$ and $Ce_{0.99}Cu_{0.01}O_2$ under CO flow. After CO exposure, the DRIFTS spectra are recorded under continuing He flow at 10 min. The peaks at 2094 cm^{-1} are assigned to CO adsorption on separated CuO_x species for $Ce_{0.95}Cu_{0.05}O_2$ sample[6,7].

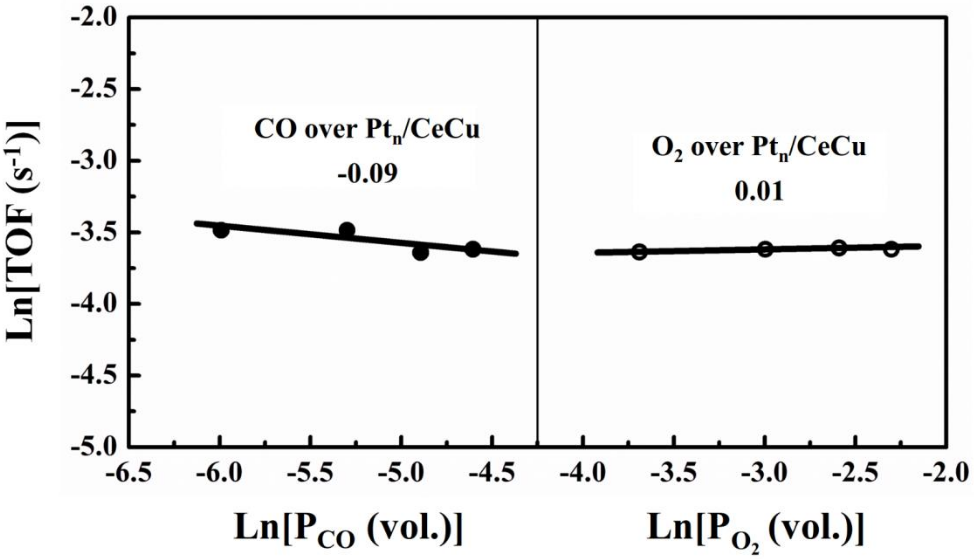

Supplementary Figure 14. Reaction orders of prepared catalyst. The reaction orders of CO and O_2 over Pt$_n$/CeCu. The feed gas was changed to 0.25~1.0% vol. CO and 2.5~10% vol. O_2 balanced by N_2 with a fixed space velocity of 120000 mL g^{-1} h^{-1}.

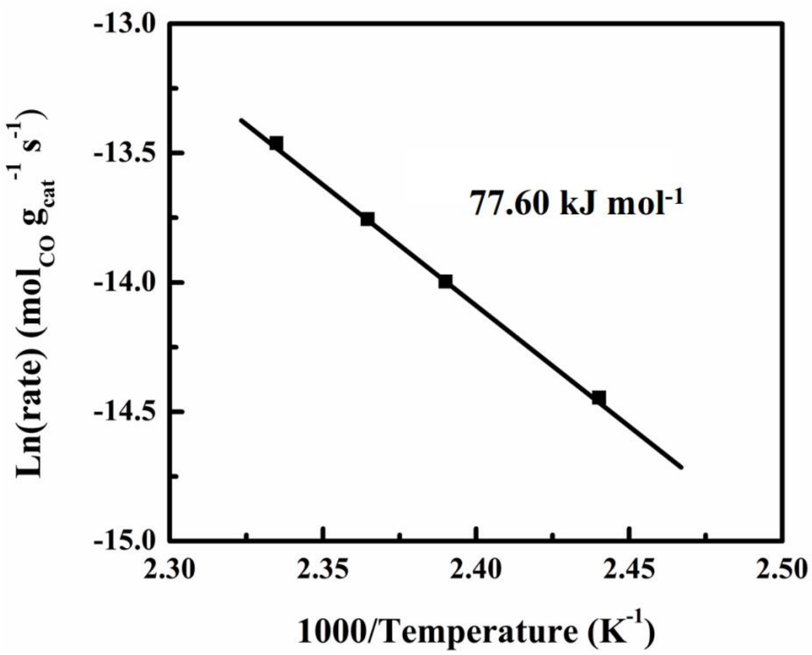

Supplementary Figure 15. Kinetic test of Cu doped CeO_2 support. Arrhenius plot of CO oxidation rate of $Ce_{0.99}Cu_{0.01}O_2$ support.

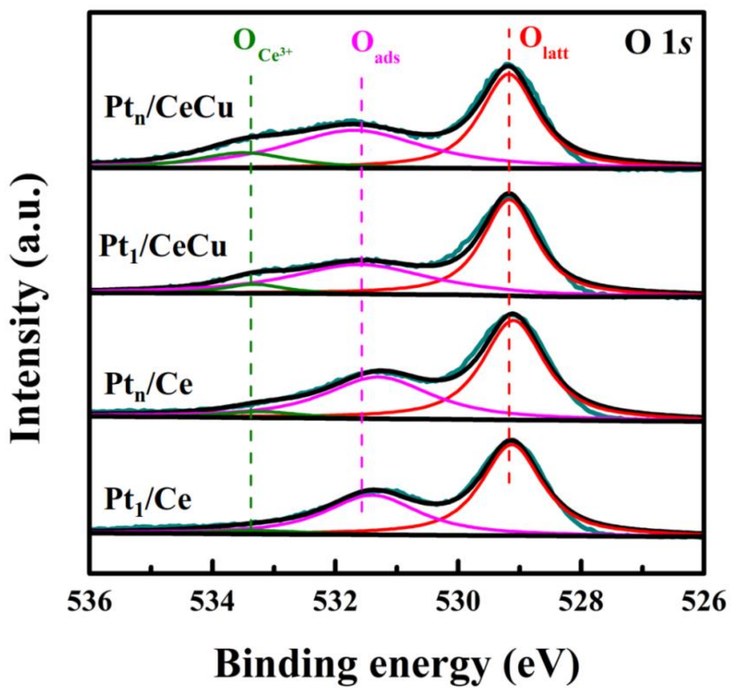

Supplementary Figure 16. Surface oxygen vacancy analysis of prepared catalysts. O 1*s* X-ray photoelectron spectroscopy (XPS) spectra of Pt_1/Ce, Pt_n/Ce, Pt_1/CeCu and Pt_n/CeCu.

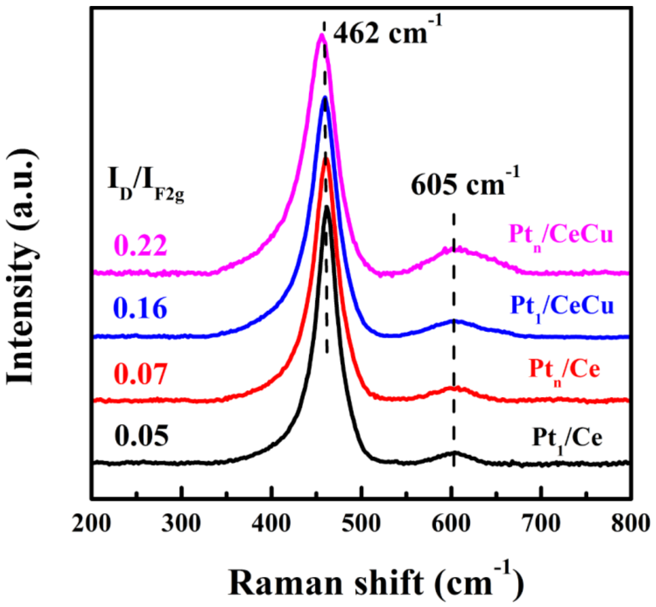

Supplementary Figure 17. Raman spectra of Pt_1/Ce, Pt_n/Ce, Pt_1/CeCu and Pt_n/CeCu. The ratios of I_D (defect-induced mode) and I_{F2g} (F_{2g} mode) have been labelled, which can imply the concentration of surface defect site.

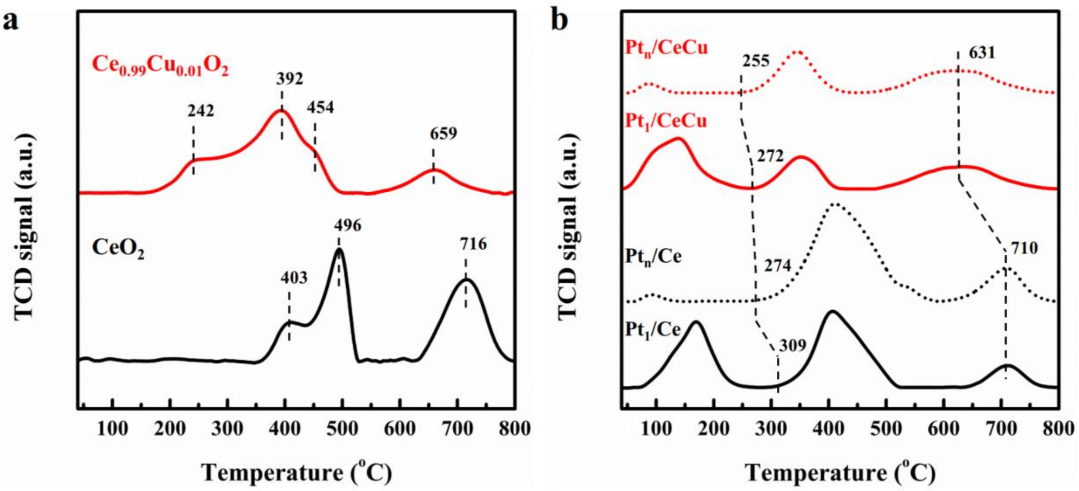

Supplementary Figure 18. Surface reducibility of prepared catalysts. Temperature-programmed reduction by hydrogen (H_2-TPR) profiles of **a** CeO_2, Cu doped CeO_2 supports and **b** Pt_n/CeCu, Pt_n/Ce, Pt_1/CeCu, Pt_1/Ce catalysts. The reduction peaks at the range of 300 ~ 550 ºC are assigned to the reduction of surface oxygen, while the peak at 716 ºC is attributed to the reduction of bulk oxygen of CeO_2 supports. The introducing of Cu can cause these reduction peaks shift to low temperature and the appearance of the reduction peak at 242 ºC can be assigned to the reduction of Cu-O-Ce species. The reduction peaks below 200 ºC are attributed to the reduction of oxidized Pt species.

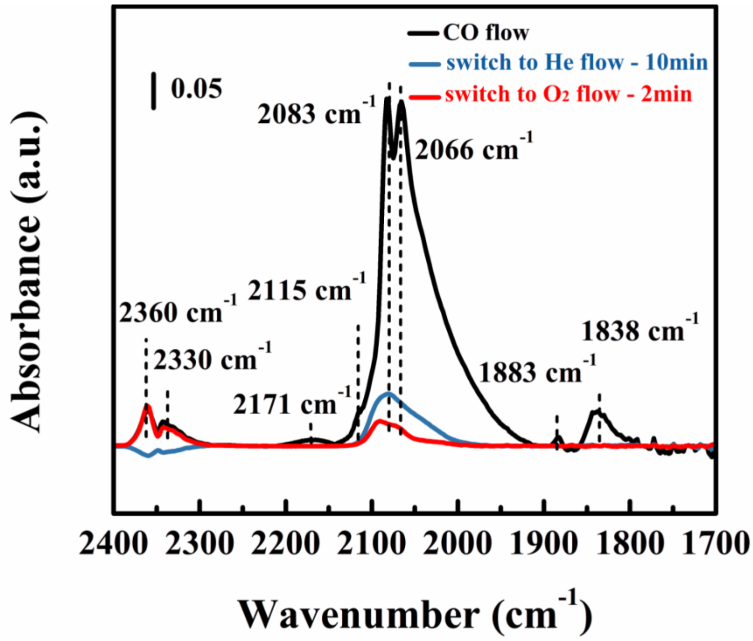

Supplementary Figure 19. *In situ* DRIFTS spectra of CO adsorption and oxidation of Pt_n/CeCu. After CO exposure, He flow is continued and the spectrum is recorded at 10 min. Subsequently, the flow is switched to 1% vol. O_2 balanced by N_2 and the spectrum is recorded at 2 min.

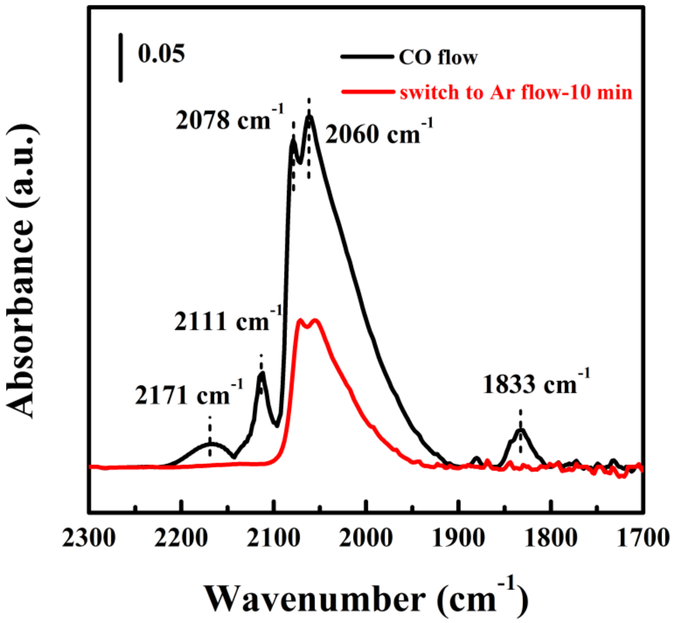

Supplementary Figure 20. *In situ* DRIFTS spectra of CO adsorption of Pt_n/CeCu. Pt_n/CeCu catalyst is pretreated at 300 ºC under 30 mL/min of 1% vol. CO balanced by Ar to deplete the active oxygen at the interfaces. After 10 min, the catalyst is cooled down to room temperature under CO flow. After CO exposure, Ar flow is continued and the spectrum is recorded at 10 min.

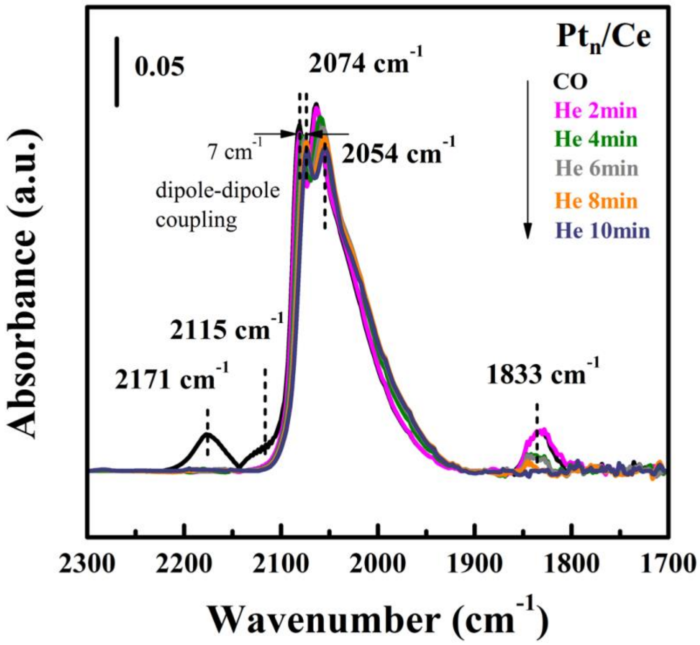

Supplementary Figure 21. *In* situ DRIFTS spectra of Pt_n/Ce under CO flow. After CO exposure, the DRIFTS spectra are recorded under continuing He flow at 2, 4, 6, 8, 10 min, which indicate the strong CO binding on CeO_2 supported Pt sub-nanoclusters. The slightly redshift of the adsorption peaks can be assigned to the changes in dipole-dipole coupling between CO molecules and Pt[8].

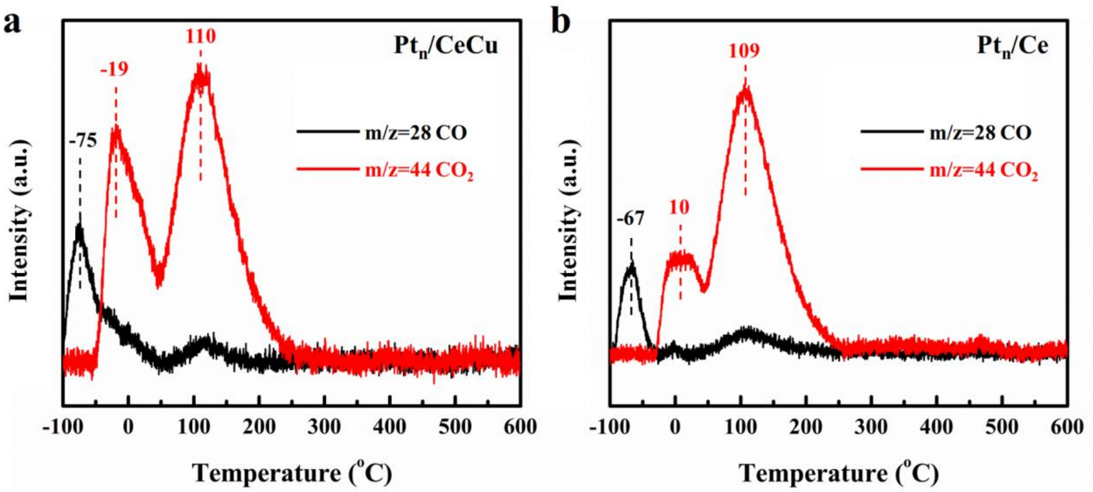

Supplementary Figure 22. CO bonding strength on supported Pt clusters. Temperature-programed desorption curves following a saturation adsorption of CO on **a** Pt_n/CeCu and **b** Pt_n/Ce catalysts at -100 ºC. The peaks at -75 and -67 ºC for Pt_n/CeCu and Pt_n/Ce can be assigned to CO desorption from Cu doped CeO_2 and CeO_2 nanorod supports, respectively, which are closely to that of Pt/CeO_2 in previous study[9]. The CO desorption and CO_2 formation at about 110 ºC for Pt_n/CeCu and Pt_n/Ce can be attributed to the activation of CO adsorbed at atop sites of Pt clusters.

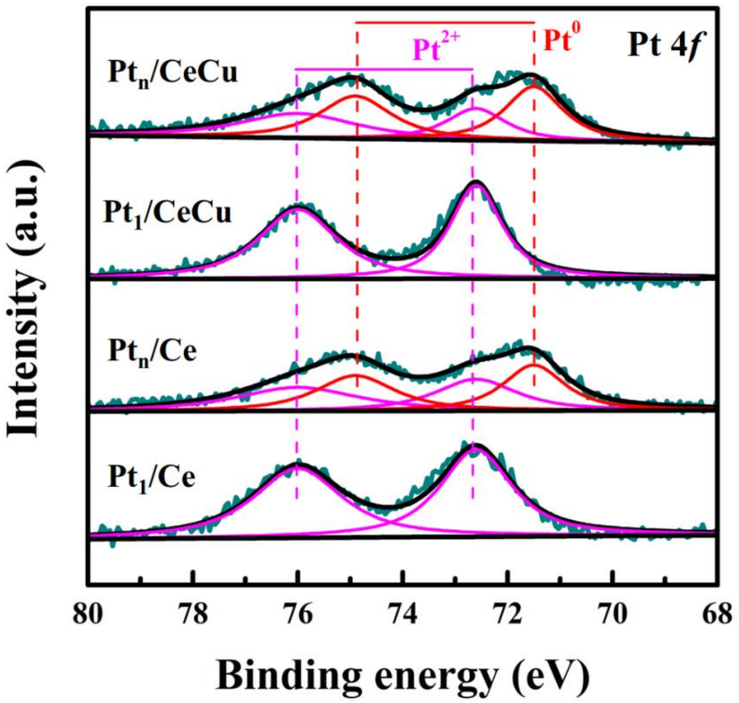

Supplementary Figure 23. Pt 4*f* XPS spectra of Pt_1/Ce, Pt_n/Ce, Pt_1/CeCu and Pt_n/CeCu.

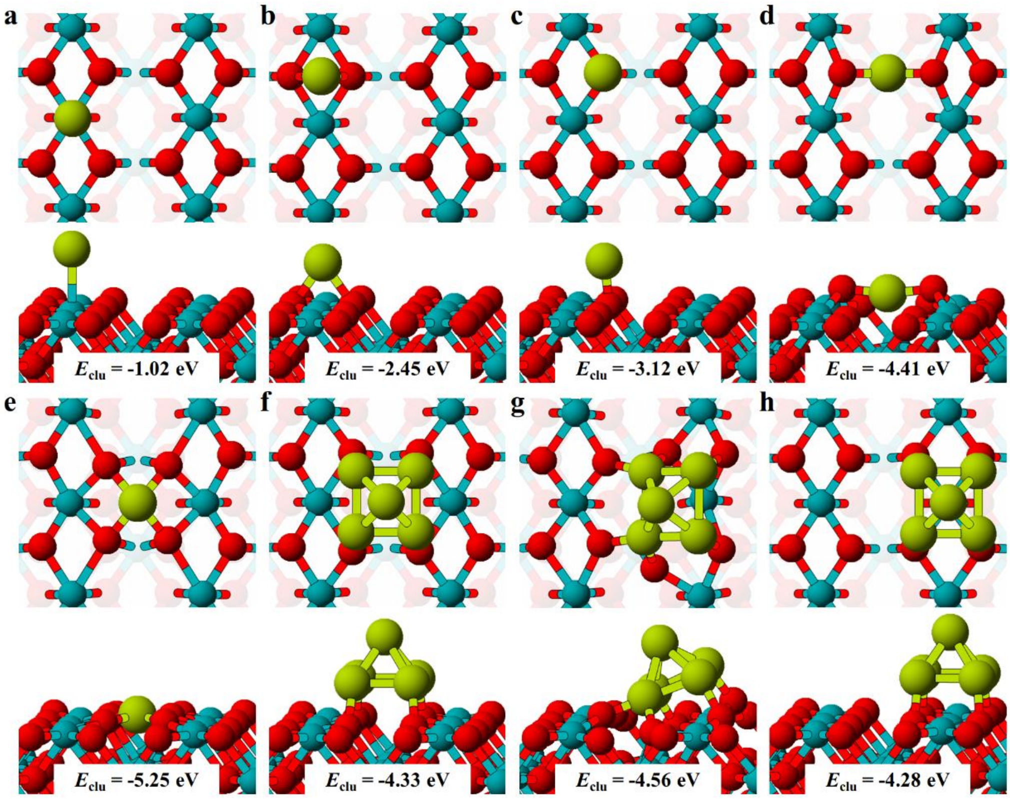

Supplementary Figure 24. Atomic models of CeO_2 supported Pt catalysts. Optimized atomic structures of **a**-**e** Pt single atom and **f**-**h** Pt_5 cluster on CeO_2 (110) slab. **e** and **g** are the most stable structures of supported Pt single atom and Pt_5 cluster with the corresponding clustering energies (E_{clu}) of -5.25 eV and -4.56 eV, respectively.

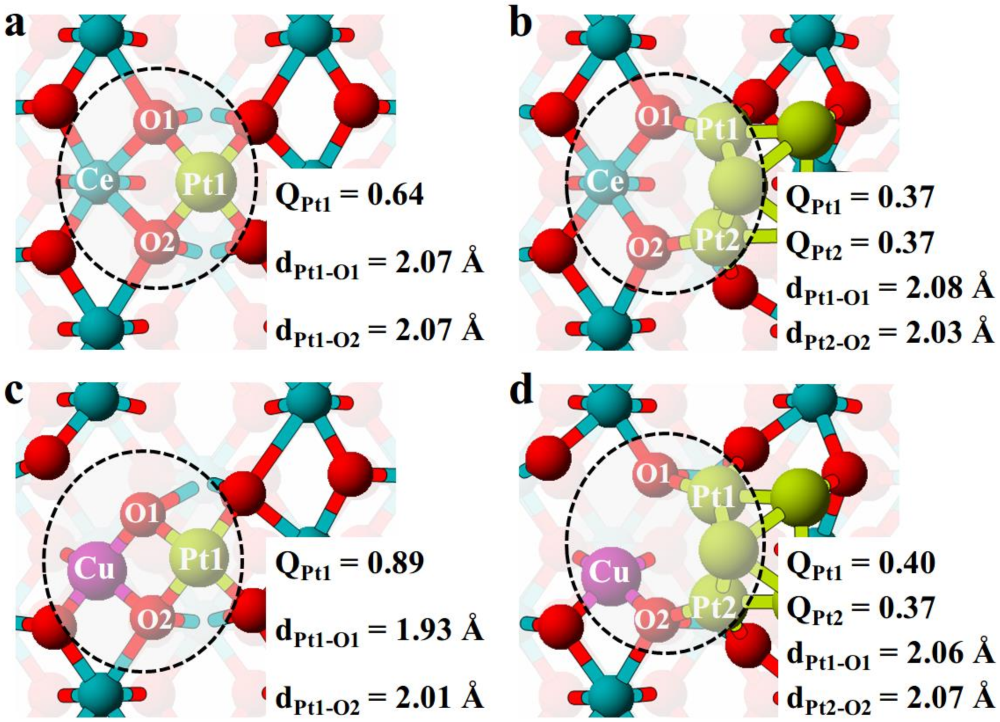

Supplementary Figure 25. Charge analysis of CeO_2 supported Pt catalysts. The most stable atomic structures of **a** Pt_1/Ce, **b** Pt_5/Ce, **c** Pt_1/CeCu and **d** Pt_5/CeCu. The corresponding Bader charges of interfacial Pt atoms and bond lengths between interfacial Pt atoms and O atoms are labelled.

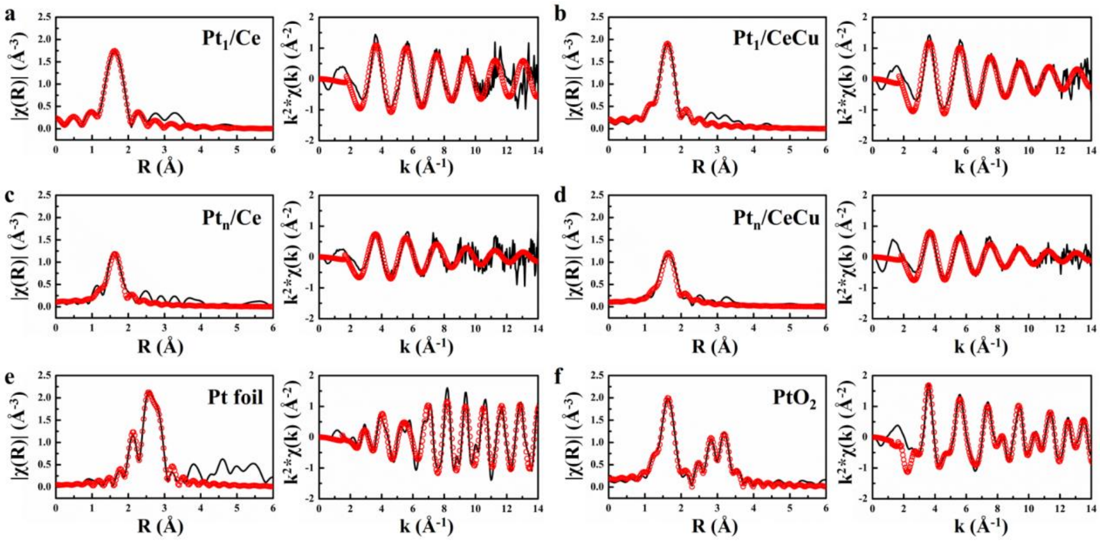

Supplementary Figure 26. Experimental (black line) and fitting (red circle) results of Fourier transformed extended X-ray absorption fine structure (EXAFS) spectra (left: R-space and right: k-space) of **a** Pt_1/Ce, **b** Pt_1/CeCu, **c** Pt_n/Ce, **d** Pt_n/CeCu, **e** Pt foil and **f** PtO_2.

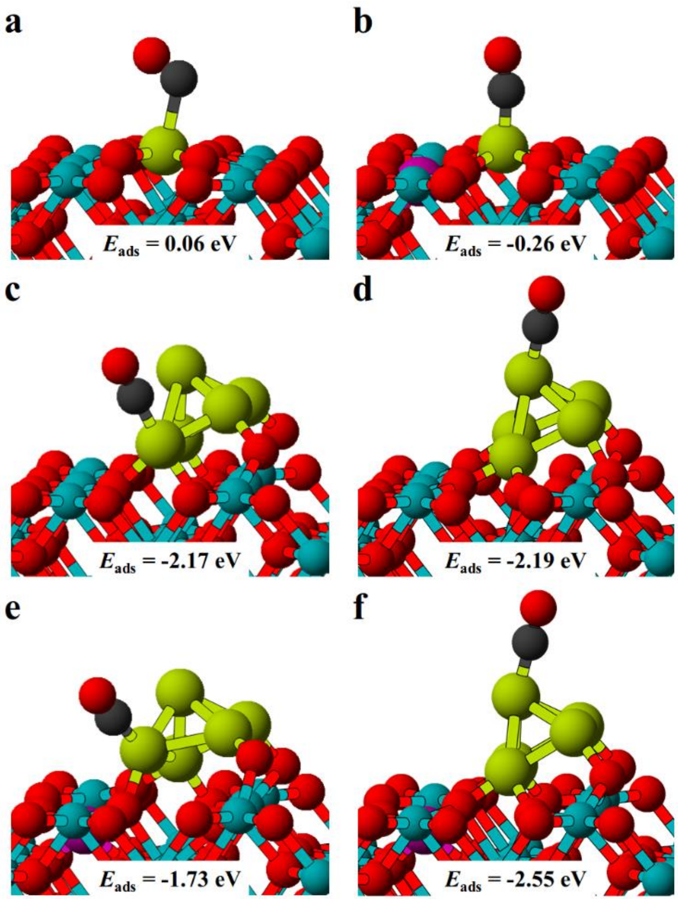

Supplementary Figure 27. CO bonding strength on CeO_2 supported Pt catalysts. Atomic structures and adsorption energies of CO adsorbed on **a** Pt_1/Ce, **b** Pt_1/CeCu, **c**-**d** Pt_5/Ce and **e**-**f** Pt_5/CeCu.

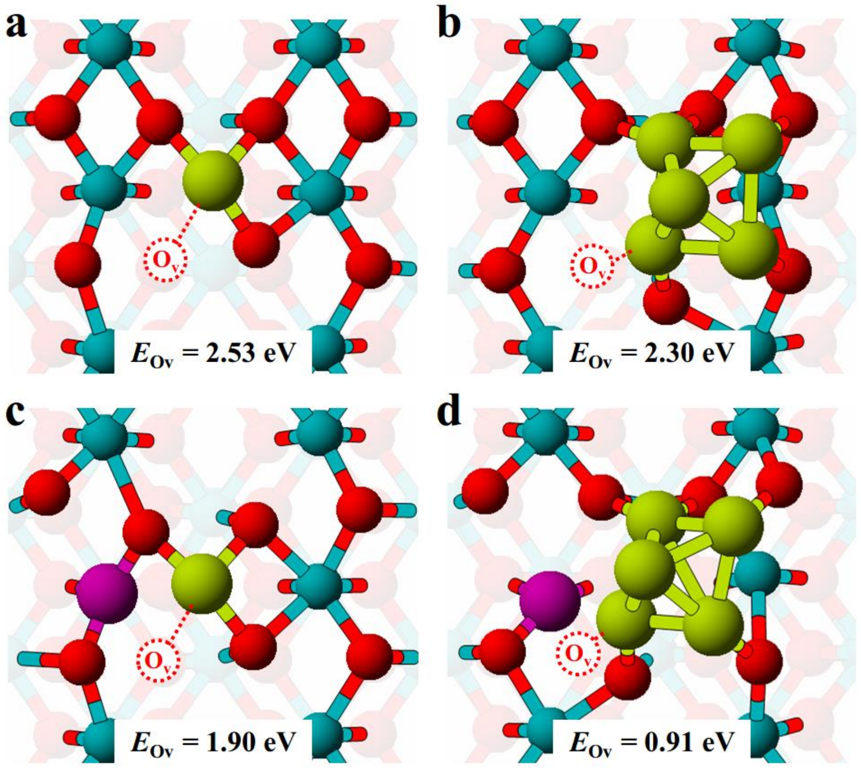

Supplementary Figure 28. Interfacial oxygen reducibility of CeO_2 supported Pt catalysts. Optimized atomic structures of **a** Pt_1/Ce, **b** Pt_5/Ce, **c** Pt_1/CeCu and **d** Pt_5/CeCu with an oxygen vacancy, respectively. The labelled oxygen vacancy formation energies (E_{Ov}s) indicate that the Cu dopants can greatly enhance the activity of interfacial oxygen.

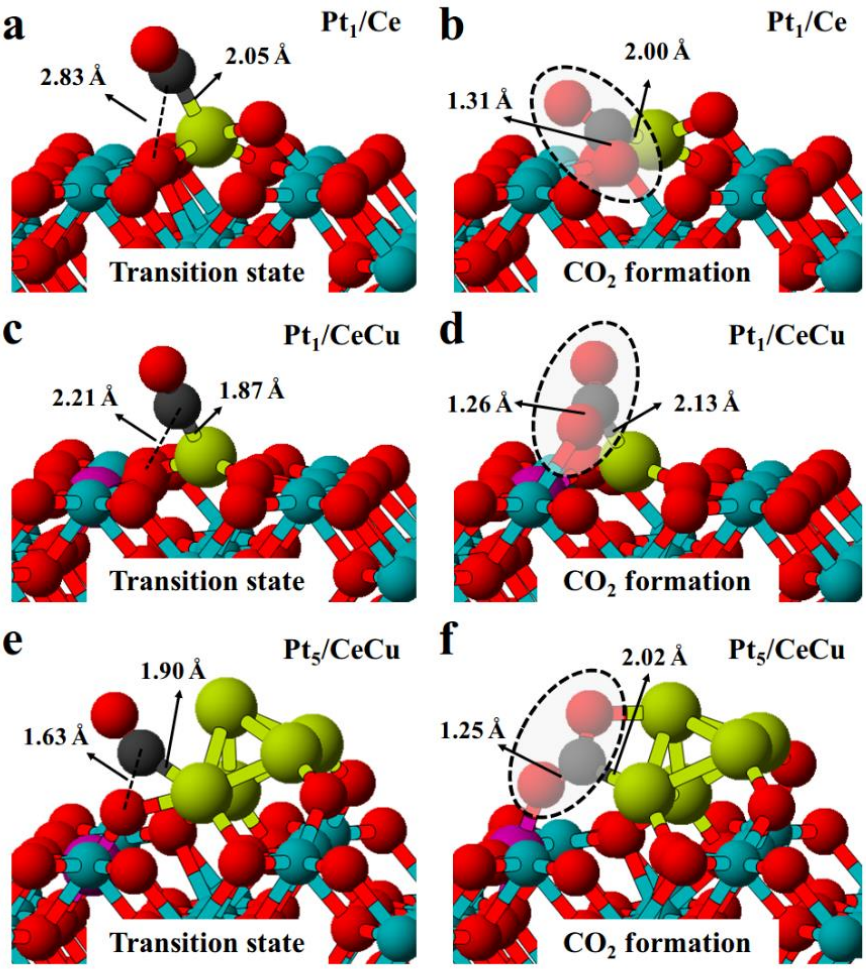

Supplementary Figure 29. Atomic structures of transition states and CO_2 formation states during CO oxidation at the interfaces of **a-b** Pt_1/Ce, **c-d** Pt_1/CeCu and **e-f** Pt_5/CeCu.

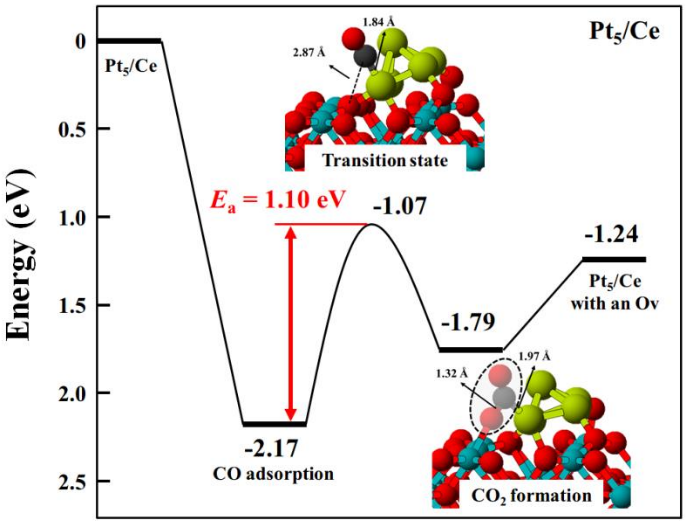

Supplementary Figure 30. Energetic route of a CO molecule oxidized by the lattice oxygen at the interface of Pt_5/Ce. The atomic structures of transition state and CO_2 formation state are inserted.

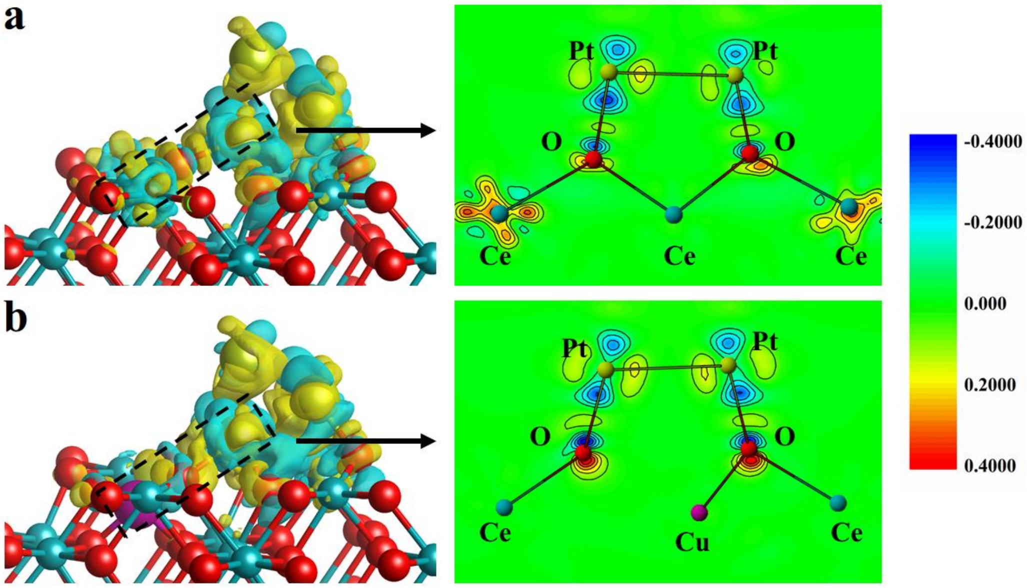

Supplementary Figure 31. Charge analysis of CeO_2 supported Pt clusters. Differential charge density ($\Delta\rho$) and the corresponding contours in the plane crossing the interfacial Pt and oxygen atoms of **a** Pt_5/Ce and **b** Pt_5/CeCu. $\Delta\rho$ is defined as $\Delta\rho = \rho_{slab+Pt5} - \rho_{slab} - \rho_{Pt5}$, where $\rho_{slab+Pt5}$, ρ_{slab} and ρ_{Pt5} are the charge densities of slab supported Pt_5 cluster, slab and an isolated Pt_5 cluster, respectively. The yellow and grey-blue represent the positive and negative charges, respectively.

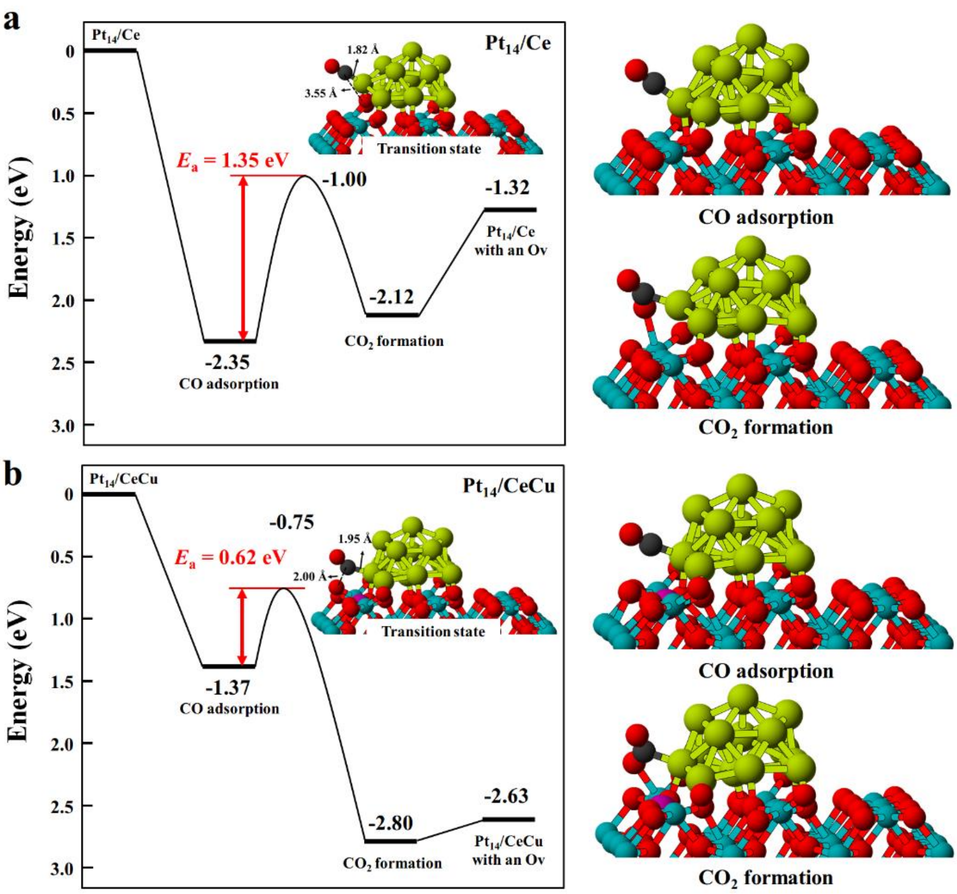

Supplementary Figure 32. CO oxidation on CeO_2 supported Pt_{14} cluster. The models of a Pt_{14} cluster on the (3 × 3) supercell of CeO_2 and Cu doped CeO_2 slabs are constructed, which are denoted as Pt_{14}/Ce and Pt_{14}/CeCu. Energetic routes of a CO molecule oxidized by the lattice oxygen at the interfaces of **a** Pt_{14}/Ce and **b** Pt_{14}/CeCu.

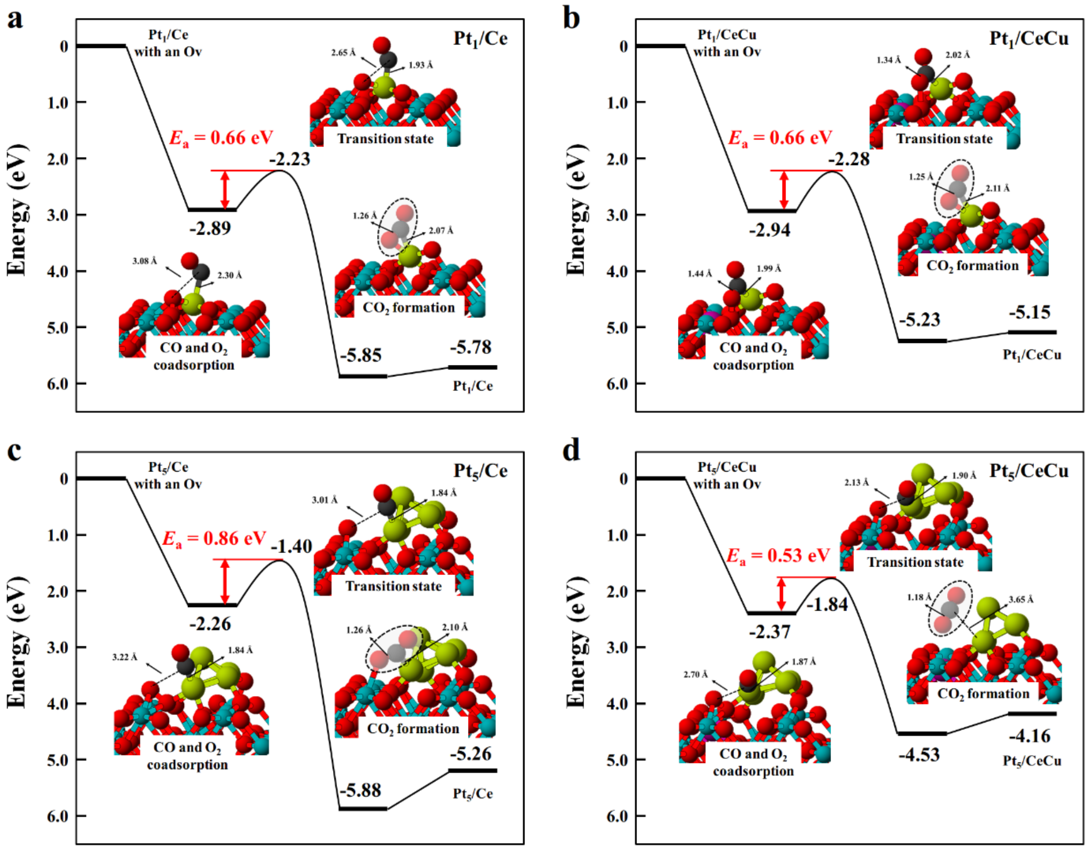

Supplementary Figure 33. CO oxidation on CeO_2 supported Pt catalysts. Energetic routes of a CO molecule oxidized by adsorbed O_2 molecule at the interfaces of **a** Pt_1/Ce, **b** Pt_1/CeCu, **c** Pt_5/Ce and **d** Pt_5/CeCu. The corresponding atomic structures of CO and O_2 coadsorption state, transition state, as well as CO_2 formation state are inserted. Note that, the energy sum of the last state and corresponding O_v + CO_2 (g) state (Figure 4 and Supplementary Figure 30) is -6.50 eV, which is equal to the total energy change of catalytic reaction cycle ($2CO + O_2 \rightarrow 2CO_2$).

Supplementary Tables

Supplementary Table 1. Lists of Pt average size (D_{Pt}, nm), Pt mass loading (wt%), onset temperature of CO oxidation (T_{onset}, ºC), turnover frequency (TOF, s^{-1}) and activation energy (E_a, kJ mol^{-1}) of our reported catalysts and that in previous studies. Note that the TOF is tested under the temperature in parentheses.

Sample	D_{Pt} (nm)	Loading (wt%)	T_{onset} (ºC)	TOF (s^{-1})	E_a (kJ mol^{-1})	References
Pt_n/CeCu	0.75	1.77 wt%	8	0.26 (80)	39.49	This work
Pt_n/Ce	0.63	0.72 wt%	36	0.03 (80)	97.62	This work
Pt_1/CeCu	Single atom	1.51 wt%	20	0.02 (80)	56.60	This work
Pt_1/Ce	Single atom	0.63 wt%	100	$<10^{-4}$ (80)	101.43	This work
Pt/CeO_2	1.6	0.5 wt%	/	0.2 (80) [a]	51	[10]
Pt/CeO_2	1.2	1.5 wt%	/	0.45 (80) [a]	44	[11]
Pt/CeO_2	0.5	1.3 wt%	/	0.01 (50) [b]	70	[12]
Pt/CeO_2	2.5	0.5 wt%	/	0.60 (200) [b]	63.7	[13]
Pt/CeO_2	1.68	1.0 wt%	25	0.101 (80)	30.1	[14]
Pt/CeO_2	1.0	2.8 wt%	30	1.97 (150)	40	[15]
Pt_1/CeO_2	Single atom	1.0 wt%	100	0.005 (80)	53.5	[14]
Pt_1/CeO_2	Single atom	1.0 wt%	150	0.12 (225)	57	[16]
Pt_1/CeO_2	Single atom	1.0 wt%	60	0.08 (80)	42.5	[17]
Pt_1/CeO_2	Single atom	0.22 wt%	60	/	61.5	[18]

*Note: [a] The TOFs are calculated based on the fraction of Pt atoms at Pt/CeO_2 interface. [b] The TOFs are normalized to the value per exposed surface Pt atoms.

Supplementary Table 2. The ratios of O_{ads} and O_{latt} (O_{ads}/O_{latt}), the concentrations of Ce^{3+} ($[Ce^{3+}]$), as well as the concentrations of oxidized state Pt ($[Pt^{2+}]$) and metallic state Pt ($[Pt^{0}]$) in prepared catalysts calculated based on the XPS results.

Sample	O_{ads}/O_{latt}	$[Ce^{3+}]$	$[Pt^{2+}]$	$[Pt^{0}]$
Pt_n/CeCu	0.99	0.28	0.43	0.57
Pt_1/CeCu	0.79	0.25	1.00	/
Pt_n/Ce	0.72	0.23	0.50	0.50
Pt_1/Ce	0.67	0.19	1.00	/

*Note: $[Ce^{3+}]$ is calculated by the formula:

$$[\mathrm{Ce}^{3+}] = \frac{A_{u'} + A_{u_0} + A_{v'} + A_{v_0}}{(A_{u'''} + A_{u''} + A_{u} + A_{v'''} + A_{v''} + A_{v}) + (A_{u'} + A_{u_0} + A_{v'} + A_{v_0})}$$

where A_i is the integrated area of corresponding peak in Ce 3*d* XPS spectra. Both O_{ads}/O_{latt} and $[Ce^{3+}]$ indicate that Cu dopants can greatly increase the concentrations of surface oxygen vacancy.

Supplementary Table 3. Structural information and fitting parameters obtained from Pt L_{III}-edge EXAFS spectra of catalysts.

Sample	Shell	N	R (Å)	σ^2 (Å^2)	ΔE_0 (eV)
Pt_1/Ce	Pt-O	3.81 ± 0.63	2.010 ± 0.008	0.0012 ± 0.0011	11.58 ± 1.03
Pt_1/CeCu	Pt-O	4.41 ± 0.28	2.004 ± 0.006	0.0010 ± 0.0007	10.64 ± 0.74
Pt_n/Ce	Pt-O	2.89 ± 0.37	2.009 ± 0.013	0.0018 ± 0.0015	10.37 ± 1.53
Pt_n/CeCu	Pt-O	3.06 ± 0.42	2.016 ± 0.014	0.0024 ± 0.0018	12.77 ± 1.60
Pt foil	Pt-Pt	12	2.765 ± 0.002	0.0046 ± 0.0003	9.15 ± 0.39
PtO_2	Pt-Pt	6	3.088 ± 0.008	0.0030 ± 0.0008	5.79 ± 2.20
	Pt-O	6	2.016 ± 0.006	0.0018 ± 0.0008	9.95 ± 0.85

Supplementary References

1. Zhou, K. B., Wang, X., Sun, X. M., Peng, Q. & Li, Y. D. Enhanced catalytic activity of ceria nanorods from well-defined reactive crystal planes. *J. Catal.* **229**, 206-212 (2005).

2. Mai, H. X. et al. Shape-selective synthesis and oxygen storage behavior of ceria nanopolyhedra, nanorods, and nanocubes. *J. Phys. Chem. B* **109**, 24380-24385 (2005).

3. Cybulskis, V. J., Wang, J., Pazmiño, J. H., Ribeiro, F. H. & Nicholas Delgass, W. Isotopic transient studies of sodium promotion of Pt/Al_2O_3 for the water-gas shift reaction. *J. Catal.* **339**, 163-172 (2016).

4. Kale, M. J. & Christopher, P. Utilizing quantitative in situ FTIR spectroscopy to identify well-coordinated Pt atoms as the active site for CO oxidation on Al_2O_3-supported Pt catalysts. *ACS Catal.* **6**, 5599-5609 (2016).

5. Liu, X., Wen, Y. W., Tang, Y. T., Lang, Y., Shan, B. & Chen, R. Effect of exposed facets and oxygen vacancies on the catalytic activity of $Pd_xCe_{1-x}O_{2-\delta}$ catalysts: a combined experimental and theoretical study. *Catal. Sci. Technol.* **7**, 4462-4469 (2017).

6. Chen, A. et al. Structure of the catalytically active copper-ceria interfacial perimeter. *Nat. Catal.* **2**, 334-341 (2019).

7. Wang, W. W. et al. Crystal plane effect of ceria on supported copper oxide cluster catalyst for CO oxidation: importance of metal-support interaction. *ACS Catal.* **7**, 1313-1329 (2017).

8. Allian, A. D. et al. Chemisorption of CO and mechanism of CO oxidation on supported platinum nanoclusters. *J. Am. Chem. Soc.* **133**, 4498-4517 (2011).

9. Jin, T., Okuhara, T., Mains, G. J. & White, J. M. Temperature-programmed desorption of CO and CO_2 from Pt/CeO_2. An important role for lattice oxygen in CO oxidation. *J. Phys. Chem.* **91**, 3310-3315 (1987).

10. Cargnello, M. et al. Control of metal nanocrystal size reveals metal-support interface role for ceria catalysts. *Science* **341**, 771-773 (2013).

11. Kopelent, R. et al. Catalytically active and spectator Ce^{3+} in ceria-supported metal

catalysts. *Angew. Chem. Int. Ed.* **54**, 8728-8731 (2015).

12. Ke, J. et al. Strong local coordination structure effects on subnanometer PtO_x clusters over CeO_2 nanowires probed by low-temperature CO oxidation. *ACS Catal.* **5**, 5164-5173 (2015).

13. An, K. et al. Enhanced CO oxidation rates at the interface of mesoporous oxides and Pt nanoparticles. *J. Am. Chem. Soc.* **135**, 16689-16696 (2013).

14. Pereira-Hernández, X. I. et al. Tuning Pt-CeO_2 interactions by high-temperature vapor-phase synthesis for improved reducibility of lattice oxygen. *Nat. Commun.* 10:1358 (2019).

15. Wang, H. et al. Surpassing the single-atom catalytic activity limit through paired Pt-O-Pt ensemble built from isolated Pt_1 atoms. *Nat. Commun.* 10:3808 (2019).

16. Jones, J. et al. Thermally stable single-atom platinum-on-ceria catalysts via atom trapping. *Science* **353**, 150-154 (2016).

17. Nie, L. et al. Activation of surface lattice oxygen in single-atom Pt/CeO_2 for low-temperature CO oxidation. *Science* **358**, 1419-1423 (2017).

18. Wang, C. L. et al. Water-mediated Mars-van Krevelen mechanism for CO oxidation on ceria-supported single-atom Pt_1 catalyst. *ACS Catal.* **7**, 887-891 (2017).
